# Effect of Portland Cement versus Sulphoaluminate Cement on the Properties of Blended Lime-Based Mortars Prepared by Carbide Slag

**DOI:** 10.3390/ma12071012

**Published:** 2019-03-27

**Authors:** Song Nie, Jianfeng Wang, Mingzhang Lan, Yali Wang, Qiaowei Zhang

**Affiliations:** College of Materials Science and Engineering, Beijing University of Technology, Beijing 100124, China; Mrniesong@126.com (S.N.); wangjianfeng@bjut.edu.cn (J.W.); wangyali1978@bjut.edu.cn (Y.W.); zhangqw1991@126.com (Q.Z.)

**Keywords:** mortar, carbide slag, cement, mechanical properties, drying shrinkage, capillary water absorption, porosity

## Abstract

In order to improve the properties of lime-based mortars and promote the green development of the construction industry, blended lime-based mortars were prepared by using carbide slag instead of hydrated lime, and the additions of Portland cement and sulphoaluminate cement were studied in our work. The paper focused on mechanical properties, porosity, capillary water absorption and drying shrinkage of both types of blended mortars. The chemical composition and microstructure of hydration products were investigated by X-ray diffraction (XRD) and scanning electron microscopy (SEM). The results show that sulphoaluminate cement provided more contributions to mechanical properties, capillary water absorption and early shrinkage compared to Portland cement.

## 1. Introduction

Air lime mortars have been used in construction since ancient times, which play the roles of bonding and decoration. However, after the invention of Portland cement at the 19th century, these traditional mortars were gradually replaced by cement mortars [1,2]. Cement mortars with the characters of fast setting and high mechanical strength are widely used in building projects now. In the past decade, with the ever-increasing requirement for energy conservation and environmental protection, air lime mortars have aroused interest of the construction industry. In comparison to cement production, air lime has low energy consumption and less CO_2_ emissions [3]. Air lime mortars have good water retention and plasticity. However, some problems remain with the use of air lime mortars as a building mortars, such as low mechanical strength, slow setting and hardening time, high water absorption by capacity, large shrinkage, and poor durability [4,5,6].

In this context, the idea of partial replace of air lime with Portland cement or pozzolans has been proposed to improve application of lime-based mortars. Relevant researches indicate that the porosity decrease and mechanical strength is improved as Portland cement content increases in lime-based mortars [7,8,9,10,11,12]. Pozzolanic materials rich in silica or alumina in the amorphous form have the ability to react with calcium hydroxide (Ca(OH)_2_) in the presence of water generating hydraulic products [13]. The pozzolanic materials commonly applied in lime-based mortars include natural materials (such as, metakaolin [13,14], calcined clays [15], diatomite [16], etc.) and by-products (such as, silica fume [17,18], cement kiln dust [19], ceramic wastes [20], etc.), which can improve their performance. Furthermore, the effect of admixtures on the properties of lime-based mortars was discussed. Iucolano et al. [21] found that fibers are able to increase strain capacity of lime-based mortars and reduce the cracking risk of mortars, Izaguirre et al. [5] presented that water repellent additives improve not only the water absorption characteristics but also the resistance to freeze-thaw. However, adding admixtures in mortars will bring some disadvantages, for example, fibers are harmful to the workability and increase porosity, etc.

Sulphoaluminate cement with the properties of fast setting and hardening, high early strength and good erosion resistance has been widely used in rush repair works, winter construction projects, and marine engineering. Sulphoaluminate cement used in this paper was made from low quality bauxite, and it has a low cost but a litter over of that of Portland cement. The main hydration products of sulphoaluminate cement is ettringite (AFt), shown in Equation (1), which can fill the pores of mortars and improve the mechanical properties [3,22]. In that respect, employing sulphoaluminate cement in lime-based mortars is an effective method for optimization of performance.

C_4_A_3_S + 2CSH_2_ + 36H → C_6_AS_3_H_32_ (AFt) + 2AH_3_ (1)

On the other hand, significant reutilization of industrial residue has become more popular in the construction industry. Carbide slag is industrial residue of the hydrolysis of calcium carbide to produce acetylene (C_2_H_2_), which is a raw material for the production of polyvinyl chloride (PVC) and some other industrial products [23,24], presented in Equation (2). About 1.5–1.9 t of calcium carbide slag are discharged in the production of 1.0 t of PVC [25]. In China, large quantities of carbide slag are produced every year, and the annual output of calcium carbide slag was about 20.1 million tons in 2013 [26]. The main component of calcium carbide slag is Ca(OH)_2_, which has been used in many fields, such as inorganic cementitious materials production [27,28,29,30], chemical products synthesis [31,32], as well as CO_2_ or SO_2_ capture [25,33] etc. In the past decade, some researchers proposed utilizing calcium carbide slag instead of limestone to produce cement, which can save limestone resources and reduce CO_2_ emission [29]. Based on alkaline-activated effect, carbide slag was also mixed with fly ash, granulated blast furnace slag or other potentially active materials to produce binder [34,35,36]. Nonetheless, most of carbide slag has not been effectively reused and was landfilled. It not only occupies valuable land, but also alkalizes soils and waters. Therefore, it is extremely urgent to find new disposal strategies to recycle carbide slag.

CaC_2_^(s)^ + 2H_2_O^(l)^ → C_2_H_2_^(g)^ + Ca(OH)_2_^(aq)^ (Carbide slag) (2)

In this paper, hydrated lime was replaced by carbide slag to produce lime-based mortars. This study focuses on the comparison between carbide slag-sulphoaluminate cement blended mortars and carbide slag-Portland cement blended mortars in terms of bulk density, porosity, capillary water absorption, drying shrinkage and mechanical properties. Hydration products and microstructures of both types of blended mortars were characterized by XRD and SEM.

## 2. Materials and Methods

### 2.1. Materials

Carbide slag used in this study was supplied Shaanxi Jintai Chlor-alkali Chemical Co., Ltd., Yulin, China, with a density of 2.34 g/cm^3^ and a specific surface area of 501 m^2^/kg. Portland cement (P·I 42.5 according to Chinese National Standard GB 175-2007 [37]) was provided by Shandong Lucheng Cement Co., Ltd., with a density of 3.18 g/cm^3^ and a specific surface area of 384 m^2^/kg. Sulphoaluminate cement was a commercial cement supplied by Tangshan Polar Bear Building Materials Co., Ltd., Qufu, China, with a density of 2.96 g/cm^3^ and a specific surface area of 460 m^2^/kg. Its strength grade is 42.5 according to Chinese Building Materials Industry Standard JC/T 2282-2014 [38]. The chemical composition of carbide slag, Portland cement and sulphoaluminate cement is shown in Table 1. Quartz sand is produced by Xiamen Aisiou Standard Sand Co., Ltd., Xiamen, China.

**Figure 1:**
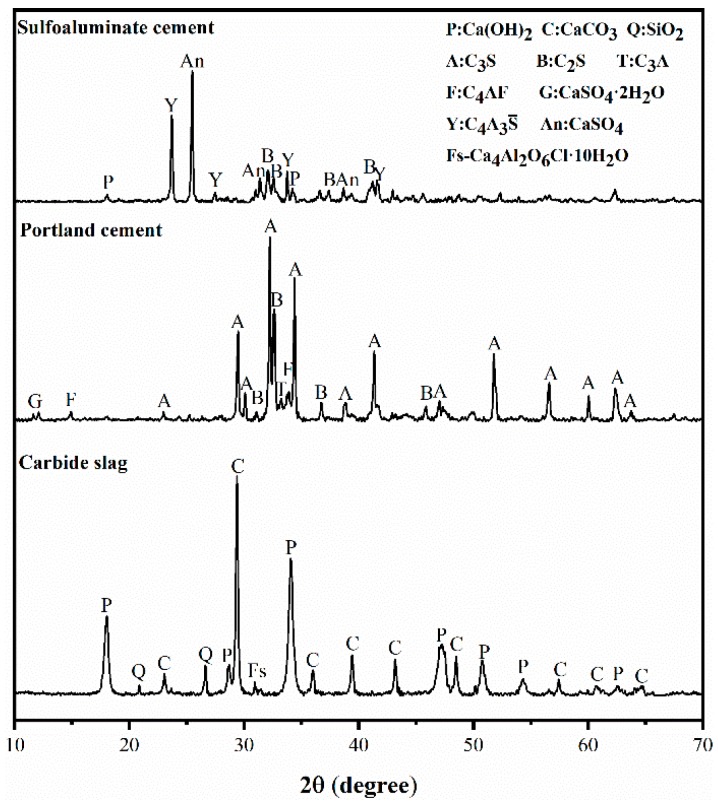
XRD patterns of raw materials used in this research.

The mineral composition of all materials was determined by X-ray Diffraction (XRD), and the results are shown in Figure 1. Carbide slag was composed of portlandite (Ca(OH)_2_), calcite (CaCO_3_), silicon dioxide (SiO_2_) and vestiges of hydrocalumite, as known as Frieded’s salt (Ca_4_Al_2_O_6_Cl·10H_2_O). XRD patterns of Portland cement revealed the presence of tricalcium silicate (C_3_S), dicalcium silicate (C_2_S), tetracalcium aluminoferrite (C_4_AF), tricalcium aluminate (C_3_A) and a little gypsum (CaSO_4_·2H_2_O). There are four mineral phases in sulphoaluminate cement, including ye’elimite (C_4_A_3_S), dicalcium silicate (C_2_S), anhydrite (CaSO_4_) and portlandite (Ca(OH)_2_).

### 2.2. Mix Design

Altogether nine different compositions of blended mortars were prepared by binder (carbide slag, Portland cement or sulphoaluminate cement) and quartz sand with a ratio of 1:3 by mass. Carbide slag was replaced by Portland cement or sulphoaluminate cement with 0% (CS), 10% (CS-10PC or CS-10SAC), 20% (CS-20PC or CS-20SAC), 30% (CS-30PC or CS-30SAC) and 40% (CS-40PC and CS-40SAC) of binder mass. All blended mortars were prepared using the amount of water to achieve a mortar slump of 160 ± 10 mm measured by the flow table test based on Chinese National Standard GB/T 2419-2005 [39], and mortars obtained a good workability. Table 2 presents the mix proportions of blended mortars.

### 2.3. Methods

#### 2.3.1. Mechanical Properties

Mechanical properties of blended mortars were evaluated by flexural strength and compressive strength in accordance with Chinese National Standard GB/T 17671-1999 [40]. Mortars were casted a prismatic mold with dimensions 4 × 4 × 16 cm and demolded after 5 days. Specimens were cured in 23 ± 2 °C and 50 ± 5% relative humidity (RH) until 28 days. The mass and volume of each prism was measured, and then bulk density was calculated. Flexural strength was determined by a three-point bending test at a loading speed of (50 ± 10) N/s, and six broken prism specimens after a three-point bending test was used to measure compressive strength test at a loading speed of (2400 ± 200) N/s. Flexural and compressive strength were taken as the average of the test results.

#### 2.3.2. Porosity

The porosity of blended mortars was determined according to EN 1936: 2006 [41]. Three pieces from the center of the mortar samples was dried in a vacuum oven at 40 °C until a constant mass was reached. The porosity (p) of is determined by means of the following Equation (3):

p = (m_s_ − m_d_)/(m_s_ − m_h_) × 100 (3)

where m_h_ is the mass of the specimen immersed in water; m_d_ is the mass of the dry specimen; m_s_ is the mass of the saturated specimen.

#### 2.3.3. Capillary Water Absorption

Capillary water absorption was performed based on EN 1015-18: 2002 [42]. Three prism specimens (4 × 4 × 16 cm^3^) were prepared, and then broken into two halves. The four long faces of the specimens were sealed by epoxy resin. All test specimens were dried under 60 °C until constant mass was reached. Dry weight of specimens was recorded. Specimens were placed in a container, with the broken faces downwards, immersed in water to a depth of 5 mm to 10 mm. The container was kept closed in order to reduce water evaporation. Specimens were weighted in 0, 10 min, 30 min, and in each hour until 8 h, and then every day until mortars reached constant mass.

Curves of capillary water absorption were determined by the square root of time and capillary water absorption mass per unit area. Capillary coefficient represents the initial capillary water absorption rate, which depends on the slope of the initial linear part of capillary water absorption curves, and asymptotic value was the maximum of water absorption per unit area during the test.

#### 2.3.4. Dry Shrinkage and Weight Loss

Dry shrinkage of blended mortars was measured according to Chinese Building Industry Standard JGJ/T 70-2009 [43]. Three prismatic specimens (4 × 4 × 16 cm^3^) were cured in a condition of 23 ± 2 °C and 90 ± 5% RH, and then demolded after 1 days, the initial length and weight were immediately recorded. Specimens were then placed in an environment of 23 ± 2 °C and 50 ± 5% RH. The length change of the specimens was measured by a dial gauge with a measurement accuracy of 0.001 mm, and the weight was also measured.

#### 2.3.5. X-ray Diffraction

The same amount of water was used to prepare paste samples. Part of samples from the center of the paste samples was dried in a vacuum oven at 40 °C for at least 24 h. The dried samples were ground and filtered using 0.056 mm sieve for X-ray diffraction (XRD) test. The XRD data was recorded using a German Bruker D8 ADVANCE X-ray diffractometer (Karlsruhe, Germany) equipped with the CuKα radiation (λ = 1.54 Å) and a fixed divergence slit size 0.02°. The 2θ range was 10–70°.

#### 2.3.6. Scanning Electron Microscopy 

The microstructure of mortars was investigated by Quanta FEG 250 field emission environmental scanning electron microscopy (SEM, FEI Company, Hillsboro, OR, America) with a low accelerating voltage (5 kV). Before SEM test, the specimens were treated by spray-gold.

## 3. Results

### 3.1. Mechanical Properties

**Figure 2:**
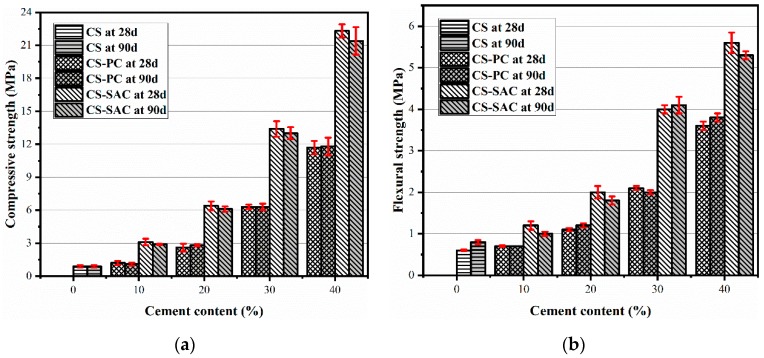
Compressive and flexural strength of blended mortars: (**a**) Compressive strength; (**b**) flexural strength.

Figure 2 gives the results of compressive and flexural strength of blended mortars at 28 days and 90 days. The compressive and flexural strength of mortars remain steady when the curing age increases from 28 days to 90 days. Carbide slag mortars have the lowest compressive and flexural strength, and both of them are less than 1.0 MPa. For CS-PC blended mortars, compressive and flexural strength have almost no change until the content of Portland cement is more than 20%, which is in agreement with Arandigoyen et al. [9] and Silva et al. [12]. Compared with Portland cement, sulphoaluminate cement is more favorable to the strength of blended mortars. When the content of sulphoaluminate cement in CS-SAC blended mortars is 10%, 20%, 30% and 40%, compressive strength at 28 days reaches 3.1 MPa, 6.4 MPa, 13.4 MPa and 22.3 MPa, respectively, which is approximately twice of that of CS-PC blended mortars with the same cement content.

The strength development of CS mortars is mainly from slow carbonation of Ca(OH)_2_. One reason of partly replacing carbide slag with cement is to improve early mechanical strength of blended mortars. Although two types of cement has the same strength grade, sulphoaluminate cement contributes more to the strength of blended mortars than Portland cement. In this case, sulphoaluminate cement is more suitable for the production of blended mortars to recycle carbide slag.

### 3.2. Bulk Density and Porosity

**Figure 3:**
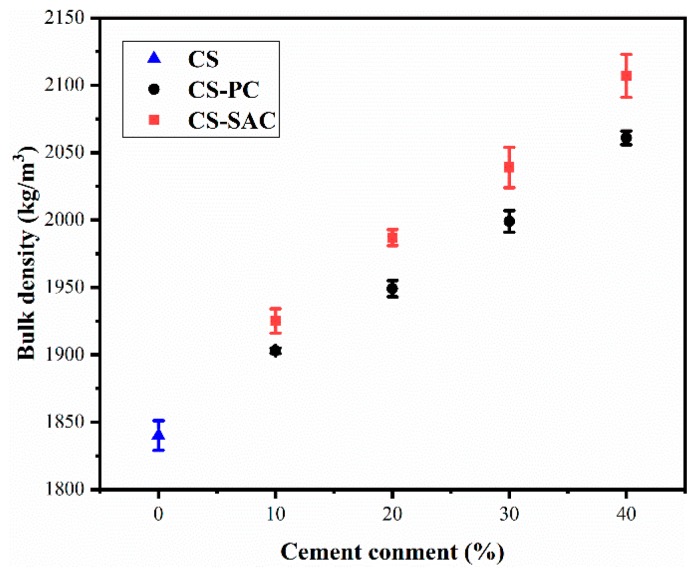
Bulk density of blended mortars at 28 days.

At 28 days of curing age (Figure 3), the bulk density of blended mortars increases from 1840 kg/m^3^ to 2061 kg/m^3^ or 2107 kg/m^3^, as the content of Portland cement or sulphoaluminate cement is 40%. Comparing two types of blended mortars, it can be indicate that the bulk density of CS-SAC blended mortars is larger than that of CS-PC blended mortars with the same cement content.

The bulk density increased because hydration products of cement tend to fill in the pore structure of blended mortars, which is consistent with SEM analysis. In addition, the water demand of blended mortars has a significant effect on the bulk density due the generation of pores when water evaporated [3].

**Figure 4:**
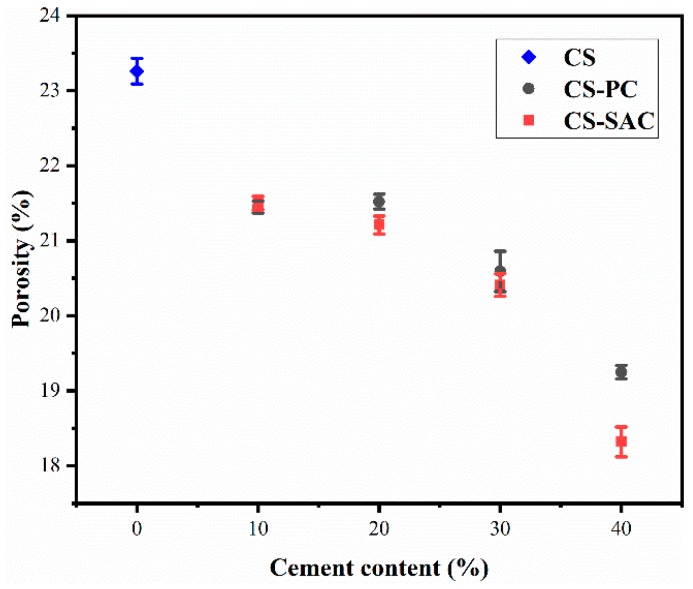
Porosity of blended mortars at 28 days.

Conversely, the porosity of blended mortars presented a decreasing trend with the increasing of cement content, especially for CS-SAC blended mortars, as shown in Figure 4. The porosity of mortars was similar to that of lime-cement mortars, which varied from 18% to 26% [12].

There are two opposite factors responsible for the change of porosity of blended mortars:

(i) A decrease of mixing water and the formation of hydration products diminish the porosity of blended mortars due to the addition of cement.
(ii) The sand volume increases with the content of cement in blended mortars (Table 3), which causes a rise of interfacial transition zone (ITZ). Therefore, the porosity in ITZ increases.

The porosity of blended mortars is approximately a constant when the content of cement increases from 10% to 20%. However, the content of cement will dominate the change of porosity as cement content over 20%.

### 3.3. Capillary Water Absorption

**Figure 5:**
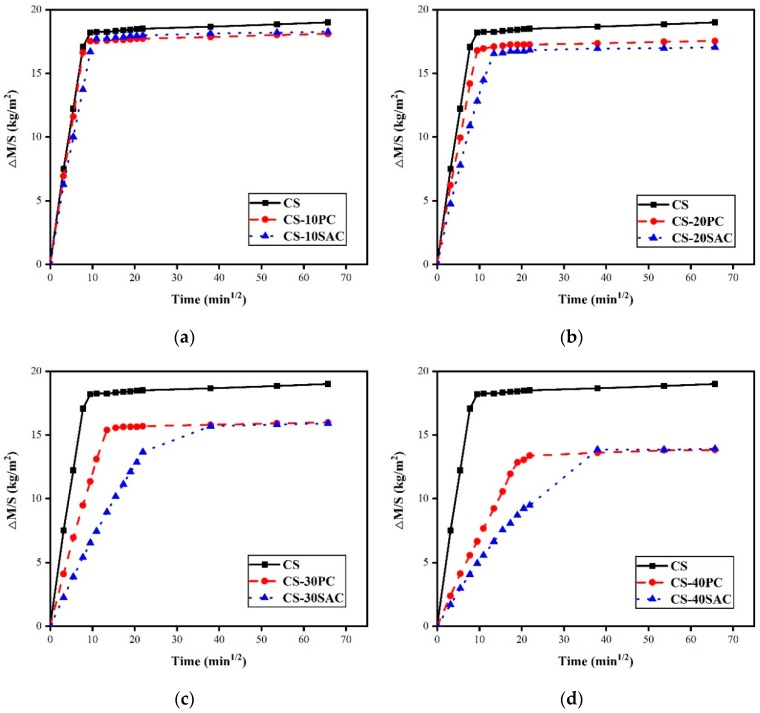
Curves of water absorption by capillarity of blended mortars at 28 days: (**a**) Blended mortars containing 10% cement; (**b**) blended mortars containing 20% cement; (**c**) blended mortars containing 30% cement; (**d**) blended mortars containing 40% cement.

Capillary water absorption is one of the main factors contributing to the durability of mortars, particularly for mortars used in building external walls. Water in the environment can penetrate into the inside of mortars by capillary force and brings about negative effects to the durability, such as, importing soluble salts and accelerate freeze-thaw cycle destruction [44]. Figure 5 presents the curves of water absorption by capillarity of blended mortars.

The initial slope of water absorption by capillarity curves of mortars without cement (CS) is the largest, and it will gradually decrease with the increase of cement content. Water absorption rate of blended mortars can be characterized by capillary coefficient. Table 4 shows the results of capillary coefficient of blended mortars. Capillarity coefficient varied from 1.72 kg·m^−2^·min^−1/2^ in mortars without cement to 0.69 kg·m^−2^·min^−1/2^ in mortars with 40% of Portland cement and to 0.52 kg·m^−2^·min^−1/2^ in mortars with 40% of sulphoaluminate cement, which reduced 58.9% and 69.8%, respectively. Apparently, sulphoaluminate cement is more conductive to the reduction of capillary water absorption of blended mortars than Portland cement. The capillary coefficient is related to water/binder ratio [45,46]. Therefore, the highest capillary coefficient of CS mortar can be explained by the highest w/c, as shown in Table 2. On the other hand, the hydration of cement tends to fill up the pores of blended mortars [8,47], which is also a reason for the reduction of capillary absorption coefficient.

In the long term, the addition of cement can decrease the maximum of water absorption (Table 4) and prolong the time water absorption reaches saturation. However, the maximum of water absorption of both types of blended mortars is similar.

### 3.4. Weight Loss

**Figure 6:**
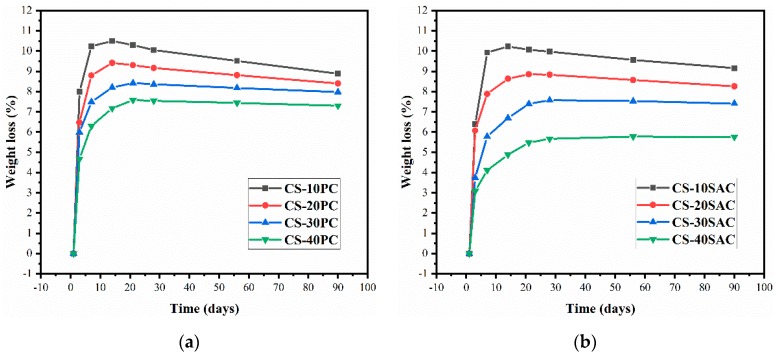
Weight loss of blended mortars: (**a**) CS-PC blended mortars; (**b**) CS-SAC blended mortars.

The weight loss of blended mortars is the result of the comprehensive effect of water evaporation and chemical reaction. Figure 6 presents the weight loss of blended mortars in 90 days. The addition of cement resulted in significantly reduction of weight loss at early ages. There are two reasons for this results: First, mixing water amount of blended mortars decreases with increasing cement content, and second, a large amount of mixing water is bound in hydration products of cement. In addition, the weight loss of CS-SAC blended mortars is significantly less than that of CS-PC blended mortars, which is related to different hydration products. The hydration product of sulphoaluminate cement is mainly AFt, which contains 32 crystal-waters. However, the main hydration product formed in Portland cement hydration is calcium silicate hydrate (C-S-H) with much less crystal water than AFt [48,49].

Almost all of blended mortars start to increase weight at time points ranging from 14 days to 28 days. At this time, the evaporation of free water has basically stopped, and carbonization reaction still takes place. The same phenomenon has been reported by Nežerka et al. [50].

### 3.5. Drying Shrinkage

**Figure 7:**
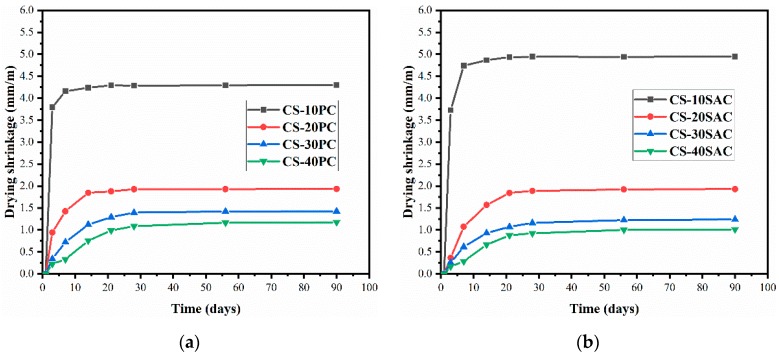
Drying shrinkage of blended mortars: (**a**) CS-PC blended mortars; (**b**) CS-SAC blended mortars.

Figure 7 presents the drying shrinkage of blended mortars. It was revealed that the addition of cement resulted in an obvious reduction of drying shrinkage, and after 28 days, blended mortars show no significant change in length. In comparison to CS-PC blended mortars, CS-SAC blended mortars have lower early shrinkage due to the faster hydration speed of sulphoaluminate cement.

The decrease of drying shrinkage of blended mortars can explain the improvement of mechanical properties. As reported by Mosquera et al. [8] micro-cracks induced by shrinkage were sharply reduced with the increasing of cement content in lime-cement mortars. When the mortars are stressed, stress concentration will occur at the micro-cracks, then the micro-cracks expand continuously and form cracks, which will lead to the damage of mortars.

**Figure 8:**
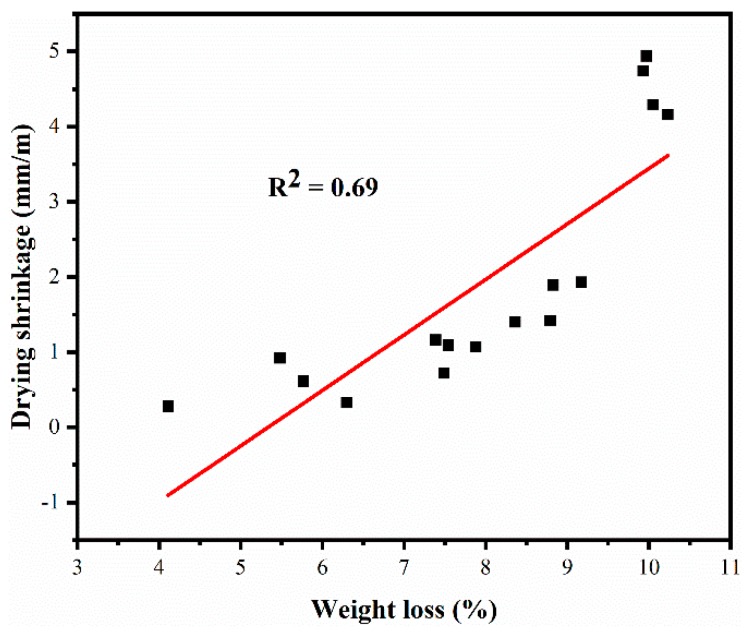
Correlation between weight loss and drying shrinkage.

The reduction of moisture in capillary pore results in the change volume of blended mortars, and weight loss can reflects the reduction of moisture in capillary pore in a way. The weight loss has a positive correlation to drying shrinkage (R^2^ = 0.69), see Figure 8. Therefore, the reduction of weight loss caused by increasing cement content in blended mortars is beneficial to reduce shrinkage.

### 3.6. X-Ray Diffraction Analysis

**Figure 9:**
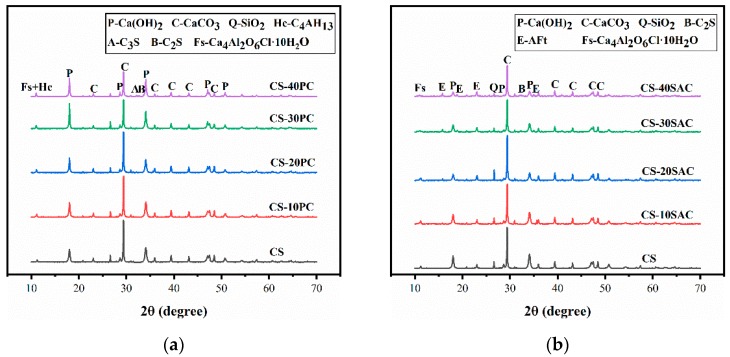
XRD patterns of blended pastes at 28 days: (**a**) CS-PC blended pastes; (**b**) CS-SAC blended pastes.

The XRD patterns of blended pastes at 28 days are presented in Figure 9. As can be seen from Figure 9a, the phase composition of CS-PC blended pastes mainly includes C_4_AH_13_, Ca(OH)_2_ and unhydrated C_3_S and C_2_S. The intensity of Ca(OH)_2_ diffraction peaks slightly increases although carbide slag content on pastes is gradually reduced, which results in the hydration of calcium silicate hydrates produce Ca(OH)_2_ and amorphous C-S-H. Compared with CS-PC blended pastes, the AFt diffraction peaks are detected in CS-SAC pastes, and their intensity is directly proportional to sulphoaluminate cement content, as shown in Figure 9b.

### 3.7. Scanning Electron Microscopy Analysis

**Figure 10:**
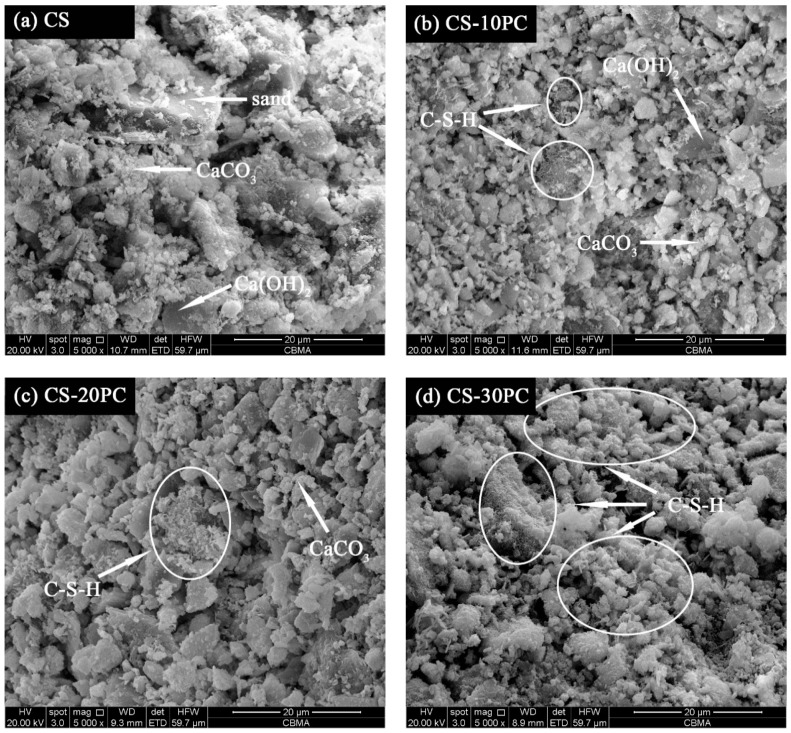

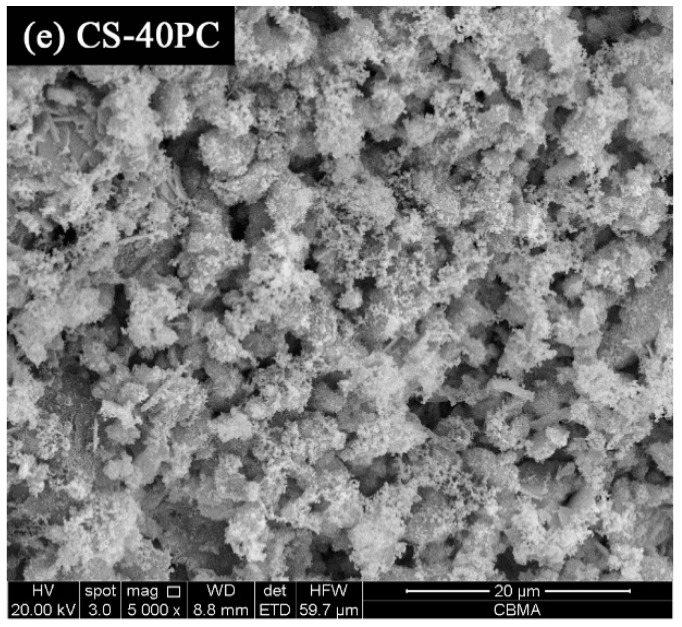
SEM images of blended mortars at 28 d: (**a**) CS; (**b**) CS-10PC; (**c**) CS-20PC; (**d**) CS-30PC; (**e**) CS-40PC.

Figure 10 displays SEM images of CS mortars and CS-PC blended mortars. Ca(OH)_2_ with hexagonal plate and CaCO_3_ particles with spherical structure can be observed in all samples. For CS mortars, tiny CaCO_3_ particles and Ca(OH)_2_ are stacked together, and the overall structure is loose, see Figure 10a. The addition of PC results in C-S-H gel can be visible in the microstructure of CS-PC blended mortars. As is depicted in Figure 10b,c, fibrous C-S-H gel with aggregate state is not evenly distributed in sample CS-10PC and simple CS-20PC. However, network C-S-H gel distributes uniformly when the content PC exceeds 20%, as shown in Figure 10d,e. C-S-H gel adheres to the surface of Ca(OH)_2_ and CaCO_3_, forming a compact microstructure. It can be explained that the strength of CS-PC blended mortars increases obviously when the content of Portland cement exceeds 20%.

**Figure 11:**
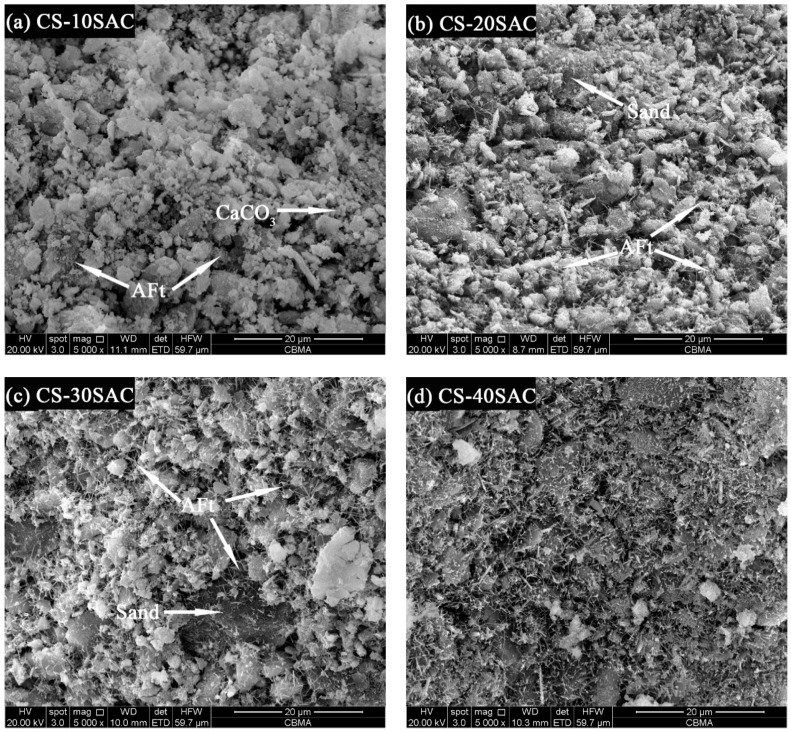
SEM images of blended mortars at 28 d: (**a**) CS-10SAC; (**b**) CS-20SAC; (**c**) CS-30SAC; (**d**) CS-40SAC.

The results of SEM images of CS-SAC blended mortars are shown in Figure 11. Besides Ca(OH)_2_ and CaCO_3_, fine-needle like AFt can also be very well visible With the increase of sulphoaluminate cement content, the amounts of AFt increases significantly. Massive AFt fills in the pores and interlaces with other phases (Ca(OH)_2_, CaCO_3_ et al.) to form a compact structure, which is conducive to improving the water absorption performance of CS-SAC blended mortars. In addition, the interlaced AFt provides a framework structure for mortars [51,52], which is good for the development of strength of mortars.

## 4. Conclusions

In this research, the effect of Portland cement and sulphoaluminate cement on the properties of blended mortars prepared by carbide slag was studied. The following conclusions could be drawn from the experiments:

- The addition of cement can significantly improve the compressive and flexural strength of blended mortars. For CS-PC blended mortars, the content of Portland cement should be more than 20% so than to enhance mechanical properties. Compared with Portland cement, sulphoaluminate cement contributes more to the strength, the compressive and flexural strength of CS-SAC blended mortars are almost 2 times stronger than that of CS-PC blended mortars.
- The addition of cement can diminish the porosity of blended mortars, which results in the increase of strength and the reduction of capillary water absorption. CS-SAC blended mortars have lower capillary coefficient than CS-PC blended mortars, but their maximum water absorption is nearly equal.
- The drying shrinkage of blended mortars effectively reduces due to the presence of cement, which is one of the reasons to improve mechanical behavior. The fast formation of AFt constrains a large amount of mixing water, which reduces the evaporation of water and enables CS-SAC blended mortars to have lower early shrinkage.
- The microstructure depends on cement type in blended mortars. C-S-H gel in CS-PC blended mortars mainly covers the surface of other phases, while fine-needle like AFt interlaces with other phases, forming a dense microstructure.

Blended mortars prepared by carbide slag have the potential to be used in masonry or plastering engineering, and the recommended cement content is more than 20% so that blended mortars have good performance.

## Figures and Tables

**Table 1:** Chemical composition of raw materials used in this research using XRF (wt%). XRF: X-ray fluorescence.

Contents	SiO_2_	Al_2_O_3_	Fe_2_O_3_	CaO	MgO	SO_3_	TiO_2_	Cl	LOI
CS	7.82	2.61	0.62	58.50	0.84	1.09	0.00	0.15	28.20
PC	20.12	5.27	2.95	64.90	2.51	1.22	0.40	0.00	1.13
SAC	16.83	17.41	1.38	44.68	4.47	12.58	0.88	0.00	0.98

CS = Carbide slag, PC = Portland cement, SAC = Sulphoaluminate cement. LOI: Loss on Ignition.

**Table 2:** Mix proportions (kg/m^3^) and workability of mortars.

Samples	CS	PC	SAC	Sand	Water/Binder Ratio	Slump (mm)
CS	450	0	0	1350	0.65	168
CS-10PC	415	45	0	1350	0.62	167
CS-20PC	360	90	0	1350	0.59	170
CS-30PC	315	135	0	1350	0.57	169
CS-40PC	270	180	0	1350	0.55	168
CS-10SAC	415	0	45	1350	0.62	167
CS-20SAC	360	0	90	1350	0.59	163
CS-30SAC	315	0	135	1350	0.57	169
CS-40SAC	270	0	180	1350	0.54	165

**Table 3:** Binder/sand ratio of mortars.

Mortars	Binder/Sand Ratio by Mass					Binder/Sand Ratio by Volume			
	CS	PC	SAC	Sand		CS	PC	SAC	Sand
CS	1.0	-	-	3.0		1.00	-	-	2.61
CS-10PC	0.9	0.1	-	3.0		0.92	0.08	-	2.68
CS-20PC	0.8	0.2	-	3.0		0.84	0.16	-	2.76
CS-30PC	0.7	0.3	-	3.0		0.76	0.24	-	2.83
CS-40PC	0.6	0.4	-	3.0		0.67	0.33	-	2.92
CS-10SAC	0.9	-	0.1	3.0		0.92	-	0.08	2.67
CS-20SAC	0.8	-	0.2	3.0		0.83	-	0.17	2.72
CS-30SAC	0.7	-	0.3	3.0		0.75	-	0.25	2.78
CS-40SAC	0.6	-	0.4	3.0		0.65	-	0.35	2.85

**Table 4:** Capillarity coefficient and asymptotic value of mortars at 28 days.

Samples	Capillarity Coefficient (kg·m^−2^·min^−1/2^)	Asymptotic Value (kg/m^2^)
CS	1.71	19.0
CS-10PC	1.70	18.1
CS-20PC	1.69	17.5
CS-30PC	1.16	16.0
CS-40PC	0.68	13.8
CS-10SAC	1.67	18.3
CS-20SAC	1.29	17.0
CS-30SAC	0.68	15.9
CS-40SAC	0.52	13.9
